# Development and implementation of the quality control panel of RT-PCR and real-time RT-PCR for avian influenza A (H5N1) surveillance network in mainland China

**DOI:** 10.1186/1471-2334-11-67

**Published:** 2011-03-16

**Authors:** Rongbao Gao, Yan Gao, Leying Wen, Ming Shao, Shumei Zou, Changgui Li, Lei Yang, Xiyan Li, Wei Wang, Yuelong Shu

**Affiliations:** 1 Dept. of Influenza, Chinese National Influenza Center, State Key Laboratory for Molecular Virology and Genetic Engineering, National Institute for Viral Disease Control and Prevention, Chinese center for disease control and prevention (China CDC), Beijing 100052, PR China; 2 The 3rd Division of Viral Vaccine, the National Institute for the Control of Pharmaceutical and Biological Products, Beijing, 100050, PR China

## Abstract

**Background:**

Reverse transcription PCR (RT-PCR) and real time RT-PCR (rRT-PCR) have been indispensable methods for influenza surveillance, especially for determination of avian influenza. The movement of testing beyond reference lab introduced the need of quality control, including the implementation of an evaluation system for validating personal training and sample proficiency testing.

**Methods:**

We developed a panel with lysates of seasonal influenza virus (H1N1, H3N2 and B), serials of diluted H5N1 virus lysates, and in-vitro transcribed H5 hemaglutinin (HA) and an artificial gene RNAs for RT-PCR and rRT-PCR quality control assessment. The validations of stability and reproducibility were performed on the panel. Additionally, the panel was implemented to assess the detection capability of Chinese human avian influenza networks.

**Results:**

The panel has relatively high stability and good reproducibility demonstrated by kappa's tests. In the implementation of panel on Chinese human avian influenza networks, the results suggested that there were a relatively low number of discrepancies for both concise and reproducibility in Chinese avian influenza virus net works.

**Conclusions:**

A quality control panel of RT-PCR and real-time RT-PCR for avian influenza A (H5N1) surveillance network was developed. An availably statistical data, which are used to assess the detection capability of networks on avian influenza virus (H5N1), can be obtained relatively easily through implementation of the panel on networks.

## Background

National and international efforts to enhance early disease detection and to increase diagnostic capacity have stimulated the formation of laboratory networks within and between public, animal, and even plant health areas. The success of these laboratory networks can be attributed to the implementation of standardized procedures and assays, specific training programs as well as a demonstrated proficiency samples. So far, avian influenza surveillance networks have formed for performance in many countries [1,2]. Highly pathogenic avian influenza virus (HPAIV) H5N1 continues to pose a significant threat to human health [3,4], although it remains a zoonotic infection [5,6]. A surveillance network with strong detection capability is required to detect any evidence that the virus has acquired the ability to transmit between humans or to emerge as the next pandemic strain.

**Figure 1:**
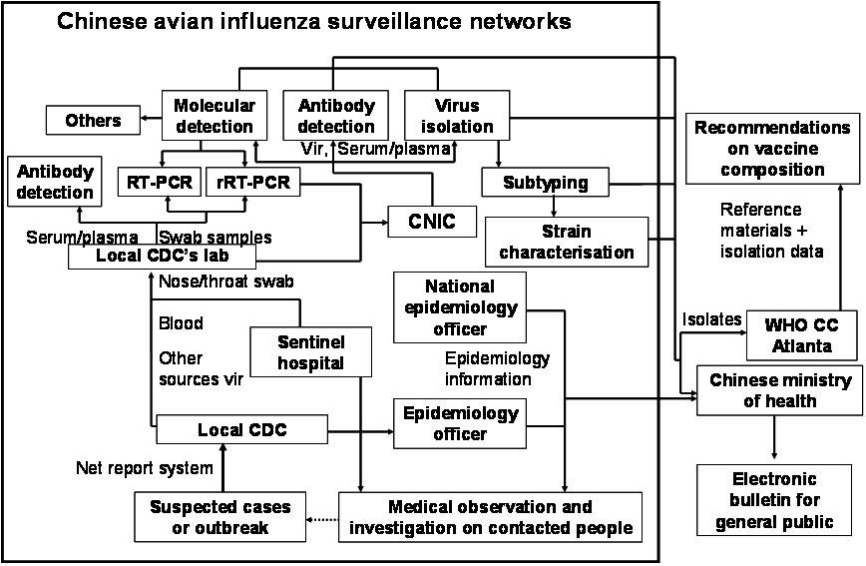
**Infrastructure and information flow of Chinese avian surveillance scheme**. Vir, virological specimens; CNIC, Chinese National influenza centre; Local CDC, local centre for disease control and prevention from China's Provinces, municipalities & Autonomous Regions; WHO, world health organization; WHO CC, WHO collaborating centre for reference and research of influenza.

The Chinese influenza surveillance scheme aims to reduce the burden of disease associated with influenza in China by collecting and exchanging timely information on influenza activity. It provides relevant information about influenza to health professionals and the general public, and contributes to the annual determination of the influenza vaccine content and to Chinese influenza pandemic preparedness activities. Compared with seasonal influenza surveillance in China, Chinese avian influenza surveillance networks have an independent infrastructure and information flow (Figure 1). Chinese national influenza centre (CNIC), that was established in 1957 and was designed as the 5^th ^WHO collaborating centre (WHO-CC) for reference and research of influenza in 2009 [7], would perform final confirmation for each suspected case in mainland China. The local laboratories of Chinese avian influenza surveillance networks could need more experiences in laboratory activities in addition to following reasons: So far, avian influenza H5N1 viruses isolated from human still are highly pathogenic [8,9]; Documented studies suggested that the virus has potent ability of human-to-human transmission [10], and the pandemic threat from highly pathogenic avian influenza viruses (HPAIV) H5N1 has not been diminished [11,12].

It is common knowledge that reliable detection techniques are necessary for influenza surveillance. Conventional methods for the detection of influenza viruses are virus isolation through inoculating samples into embryonated hen egg or by cell culture, and following to do HA and/or NA subtyping by serological methods (e.g., hemagglutinin inhibition (HI) assay). However, it should be a big limitation to emergency cases happened since an incubation period up to 1-2 weeks is often needed to achieve the subtype information later [13], and not all network laboratories meet the certificated requirements for HPAIV isolation. Molecular methods, specifically nucleic acid assay methods such as RT-PCR and rRT-PCR with high sensitivity and specificity have had indispensable effect on laboratory rapid diagnosis of HPAIV. So far, almost all influenza networks in China have developed and applied PCR detection system. However, PCR as a diagnostic tool requires a high degree of technological expertise including operation skill and procedure, environment support in addition to primers/probes and reagents. Therefore, quality control assessment (QCA) will be required to assess network performance. In this study, we developed a quality control panel for avian influenza A (H5N1) RT-PCR and rRT-PCR, and used the panel to implement assessment on avian influenza laboratories of local CDC from China's Provinces, Autonomous Regions & Municipalities.

## Methods

### Viruses

The viral strains used in this study include endemic seasonal influenza viruses (H1N1, H3N2, B) of China and avian influenza virus H5N1 (A/Guangdong/1/2005). The viruses were propagated in embryonated eggs. Virus titers were tested using hemagglutinin assay with turkey red blood cells.

### In-vitro transcribed RNA synthesis

In vitro transcribed hemaglutinin (HA) gene RNA of A/Anhui/1/2005 (H5N1) was used to determine the detection limit of the assay. The entire gene HA was synthesized and cloned into vector pSC-B (Stratagene, USA) according to the manufacturer's instructions. Gene sequence was confirmed by ABI Prism 3730 sequencer (Applied Biosystems, USA). The plasmid with T7 promoter was linearized by restriction enzyme Sac-I and then purified using DNA clean-up kit. DNA concentration was measured as OD units at 260 nm. One μg of linearized plasmid DNA was transcribed using Riboprobe in vitro transcription system kit (Promega, USA) from the T7 promoter according to the manufacturer's instructions. The transcribed RNA was purified using phenyl/chloroform solution and was quantified by spectrophotometer. RNA copy number was then determined following the method of Fronhoffs [14].

### Preparation of internal positive control (IPC) RNA

To control the possible cross-contamination in the progress of RT-PCR, an artificial gene with modified H5 HA segment which can be amplified by RT-PCR primers H5 (Table 1) was involved into the panel. The artificial gene is synthesized by inserting a 138 bp outlying gene into the middle of an H5 HA gene segment, and can be easily identified if RT-PCR amplification is performed on both segments using the same primer set as mentioned below. Therefore, the gene can be as IPC. The IPC gene segment was inserted into pGEM-T easy vector (Promega, USA) to make in vitro transcribed IPC RNA.

**Table 1:** Strength of agreement responded to value of κ

Value of κ	Strength of agreement
<0.20	Poor
0.21-0.40	Fair
0.41-0.60	Moderate
0.61-0.80	Good
0.81-1.00	Very good

### RT-PCR and rRT-PCR

The primers/probes of RT-PCR and rRT-PCR followed WHO released primer/probe sets for lab diagnosis on of HPAI H5N1 [15]. RT-PCR using QIAGEN OneStep RT-PCR kit (QIAGEN, Germany) was performed to amplify Matrix (M), Hemagglutinin (HA) and neuraminidase (NA) gene of avian influenza virus H5N1, respectively. The reaction is completed in total volume of 25 μl with 10 pM primer. The reaction mixture was incubated with 5 μl RNA at following temperature cycles. Firstly, the reverse transcription reaction was finished by 1 cycle at 60°C for 1 min, 42°C for 10 min, and 50°C for 30 min. Gene targets was then amplified by 1 cycle at 94°C for 15 min and 35 cycles at 94°C for 30 s, 52°C for 30 s and 72°C for 1 min each, and 1 cycle at 72°C for 5 min respectively. The amplification products were arrayed on 1.5% electrophoresis agarose gel. The sizes of target genes are 210 bp, 219 bp and 615 bp corresponding M, HA and NA gene, respectively. rRT-PCR for identification of all influenza A (FluA) and H5 influenza subtyping (H5) was performed using a fluorescently labeled TaqMan probe to enable continuous monitoring of amplicon formation. Primer and probe concentrations were 40 pM and 10 pM, respectively. The reaction is completed in total volume of 25 μl performed by QuantiTect Probe PCR Kit (QIAGEN, Germany). The reaction mixture was incubated with 5 μl RNA at following temperature cycles. Firstly, the reverse transcription reaction was finished by 1 cycle at 50°C for 30 min. Gene targets were then amplified by 1 cycle at 94°C for 15 min and 45 cycles at 94°C for 15 s, 55°C for 30 s and 72°C for 30 s each.

### Combination of the panel

The panel was designed to include two groups: virus lysates and in vitro transcribed RNA. The viruses were lysised in biosafety level-2 (for seasonal influenza viruses) or -3 (for avian influenza virus H5N1) containment laboratory using lysis buffer RLT (QIAGEN, Germany) as described in the manufacturer's instructions. The virus lysate group was comprised of one viral each of seasonal influenza viruses (H1N1, H3N2 and type B) and six vials of 10-fold diluted H5N1 virus (including four vials of detectable samples and two vials of undetectable samples according the pretests). The in vitro transcribed RNA group included six vials of 10-fold diluted H5 HA and one vial of IPC. Both groups used one viral sample of water as a blank control. Each viral covered enough sample for twice tests with all primers/probes as mentioned above. To ensure the consistency of the samples, 150 aliquots for each sample were prepared. Unique ID numbers were assigned to each sample to allow for single blinded detection.

### The evaluation and implementation of the panel

**Figure 2:**
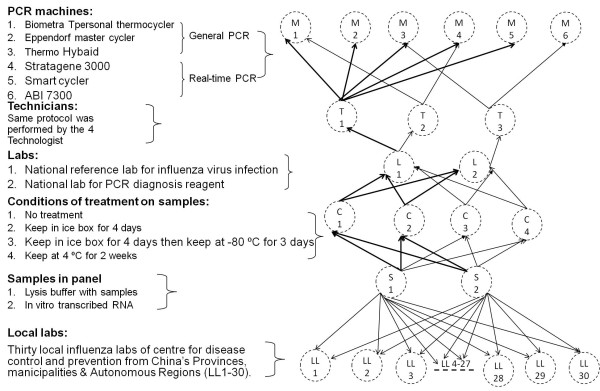
**Flow of validation and implementation for the RT-PCR and rRT-PCR panel**.

As the flow of evaluation and implementation shown in Figure 2, serial detection was performed to evaluate the panel's reproducibility. The panel was subjected to four different temperature conditions: normal storage at -80°C, 4 days in an ice box, 4 days in an ice box followed by 3 days at -80°C, and 2 weeks at 4°C. The panels with different treatments were then detected by independent technicians from two Chinese national reference laboratories (CNIC and the Chinese national reference laboratory for PCR diagnostic reagent) using the RT-PCR and rRT-PCR methods previously outlined. All detections were completed under the single blinded method. To validate the Chinese Local CDC's capability of identifying human avian influenza virus infection by RT-PCR or rRT-PCR, the aforementioned panel was implemented in 30 Local CDC laboratories (LL1~30) from China's Provinces, Municipalities & Autonomous Regions. These labs that are members of the Chinese influenza surveillance network utilized the same reagents and protocols as the Chinese National Reference Laboratories. The panel and reagents were transported to the labs by FedEx. A request was made to have all data submitted to the Chinese national influenza center before the proposed deadline date. The detections were required finished under single blinded method with a designated protocol.

### Statistical method

Kappa's tests were performed to determine the inter-rater agreement between variable technicians, PCR machines and laboratories, and between CNIC and local labs as well using SPSS statistics 17.0 software. The kappa coefficient was introduced by Cohen [16] as a chance-corrected index of agreement (CCIA) between categorical variables. The *K *value can be interpreted as Table 1[17].

## Results

### Description of the panel as RNA reference

**Figure 3:**
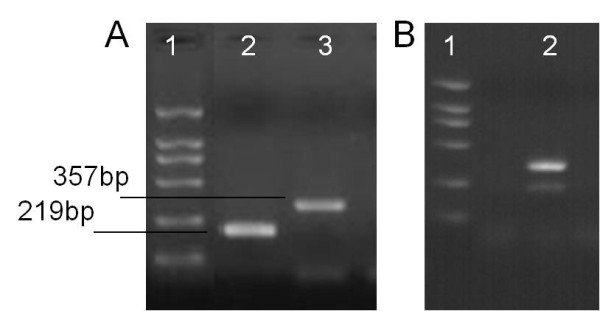
**The results of the RT-PCR for H5N1 HA and artificially modified HA gene**. A-1/B-1: DL2000 molecular weight marker; A-2: HA amplicon of H5N1 virus HA gene; A-3: Amplicon of IPC gene; B-2: Typical IPC amplificons under cross contamination happened.

The panel is designed to validate and enhance Chinese local lab of avian influenza network's ability in determination of the HPAI H5N1 virus since nucleic acid detection is the only available method for the determination of suspected H5N1 case in present local CDC of China. To validate the extraction and/or PCR procedure in the progress of detection, virus lysate and in vitro transcribed RNA were introduce into the panel. Additionally, an artificially modified HA gene segment, which can be amplified into bigger segments than viral gene in RT-PCR (Figure 3A), was integrated into the in vitro transcribed RNA to function as internal positive control (IPC). This IPC allowed for validation of cross contamination since it is common to present two bands in gel product under cross-contamination happened (Figure 3B). H2O was utilized as blank control for both lysates and in vitro transcribed RNA samples. The samples for each panel were prepared in duplicate to assess self-reproducibility. In total, 36 vials of uniquely coded sample were included in every panel.

### Validation of detectable samples in the panel

We performed detection on 8 panels selected at random to validate the detectable samples in the panel. As shown in Table 2, H5N1-1 and H5N1-2 lysates were detectable in each assay by the both RT-PCR and rRT-PCR. In vitro-transcribed RNA H5-1~H5-3 was detectable in each assay by H5 primer sets of both RT-PCR and rRT-PCR. H5N1-3 and H5N1-4 lysates combined with in-vitro transcribed RNA H5-4 were detectable in part of assays, which can be called "gray zone" samples (GZS). H3N2 and H1N1 lysates were positive just by Flu A of rRT-PCR. IPC was positive by H5 of RT-PCR only. All remaining unmentioned samples were undetectable by both RT-PCR and rRT-PCR.

**Table 2:** the sensitivity validation of the quality control system

Variation	RT-PCR			rRT-PCR	
		
	AM	H5	N1	FluA	H5
Virus lysate					

H5N1-1	8/8^a^	8/8	8/8	7/7	7/7
H5N1-2	8/8	8/8	8/8	7/7	7/7
H5N1-3	8/8	8/8	2/8	5/7	5/7
H5N1-4	3/8	1/8	1/8	1/7	2/7
H5N1-5	0/8	0/8	0/8	0/7	0/7
H5N1-6	0/8	0/8	0/8	0/7	0/7
H2O	0/8	0/8	0/8	0/7	0/7
H3N2	0/8	0/8	0/8	7/7	0/7
H1N1	0/8	0/8	0/8	7/7	0/7
FluB	0/8	0/8	0/8	0/7	0/7

In vitro transcribed RNA					

H5-1	0/8	8/8	0/8	0/7	7/7
H5-2	0/8	8/8	0/8	0/7	7/7
H5-3	0/8	8/8	0/8	0/7	7/7
H5-4	0/8	7/8	0/8	0/7	3/7
H5-5	0/8	0/8	0/8	0/7	0/7
H5-6	0/8	0/8	0/8	0/7	0/7
IPC	0/8	8/8	0/8	0/7	0/7
H2O	0/8	0/8	0/8	0/7	0/7

^a^Positive tests/total tests

### Validation of applicability for the panel

To validate the applicability of the panel, we performed serials of parallel detections on the panel including between different technicians, PCR machines, treatment conditions, and between 2 national reference laboratories as well. Kappa's tests were performed to analyze the CCIA between the detection results with varying factors. As shown in Table 3, CCIAs showed to be very good between varying factors in both RT-PCR and rRT-PCR (*k*>0.81) except a good rRT-PCR CCIA between conditions C1 and C4 (*k *= 0.804). Additionally, all of results showed completely matching (*k *= 1) between general PCR machines (M1~3), treatments on panel (C1~3) and laboratories (lab1~2).

**Table 3:** The comparison of reproducibility between technicians, machines and conditions changes

Comparison	RT-PCR		rRT-PCR	
		
	n	Kappa ± SE^a ^	n	Kappa ± SE
Technician				

T1&T2	132	0.976 ± 0.022	129	0.908 ± 0.040

Machines				

M1&M2	88	1.000 ± 0.000	/	/
M1&M3	88	1.000 ± 0.000	/	/
M4&M5	/	/	72	0.870 ± 0.073

Conditions				

C1&C2	64	1.000 ± 0.000	64	1.000 ± 0.000
C1&C3	64	1.000 ± 0.000	64	1.000 ± 0.000
C1&C4	64	0.925 ± 0.052	64	0.804 ± 0.083

Labs				

Lab1 & Lab2	84	1.000 ± 0.000	72	1.000 ± 0.000

^a^SE is the abbreviation of Standard Error.

### Implementation of the panel in local lab of Chinese influenza net work

**Figure 4:**
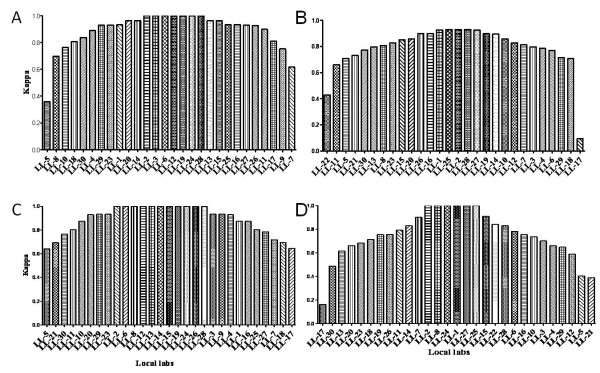
**The distribution of local labs' CCIA**. A and B diagrams present RT-PCR and rRT-PCR between local laboratories and CNIC without GZS, respectively; C and D diagrams present the RT-PCR and rRT-PCR self-reproducibility Kappa coefficient of local laboratories, respectively.

All of the implementation data were obtained before the proposed deadline date with the exception of RT-PCR results of LL22 and rRT-PCR results of LL9. The Kappa's tests were used to analyze the CCIA of the tests without GSZ between CNIC and local labs, and of local labs' self-reproducibility in duplicates. The results suggested that the CCIAs presented parabola-like distribution not only between CNIC and local labs but also in self-reproducibility (Figure 4), and RT-PCR has better CCIA in both cross concordance and self-reproducibility than rRT-PCR (Table 4). Most of laboratories presented good or very good CCIA in both RT-PCR and rRT-PCR. However, discrepancies in concordance and reproducibility were still observed. One lab each (3.45%) presented with fair and moderate RT-PCR CCIA in the cross concordance. Additionally, one lab each (3.45%) responded to poor and moderate rRT-PCR CCIA, respectively. And one (3.45%), two (6.9%) and two (6.9%) of 29 labs presented poor, fair and moderate rRT-PCR CCIA in self-reproducibility, respectively.

**Table 4:** The local labs' CCIA

Strength of agreement	Cross concordance between Local lab and CINC		Self-reproducibility	
		
	RT(%)	rRT(%)	RT(%)	rRT(%)
Poor	0 (0)	1 (3.45)	0 (0)	1 (3.45)
Fair	1 (3.45)	0 (0)	0 (0)	2 (6.90)
Moderate	1 (3.45)	1 (3.45)	0 (0)	2 (6.90)
Good	4 (13.79)	10 (34.48)	9 (31.03)	13 (44.83)
Very good	23(79.31)	17(58.62)	20 (68.97)	11 (37.93)

## Discussion

QCA studies for laboratory diagnostics of avian influenza virus help to monitor the quality of service of the participating centers, to highlight problems in particular tests or specific laboratories, and to give assurance to those centers that perform well. The objective of the QCA task group are: (i) to organize QCAs; (ii) to prepare and distribute quality control panels; (iii) to analyze and report the results; and (iv)to organize follow-up help [18]. In this study, we developed a quality control panel of RT-PCR and rRT-PCR for avian influenza A (H5N1) surveillance networks in mainland China. The panel with relatively high stability and good reproducibility was used to implement assessment of avian influenza virus detection in 30 local CDC's lab from China's Provinces, Autonomous Regions & Municipalities. The results suggested that more than 90% Chinese local influenza labs have good capability to perform detection and identification of avian influenza virus H5N1 by RT-PCR and/or rRT-PCR.

The developed quality control panel included the samples for assessment of the specificity, sensitivity and reproducibility. RT-PCR or rRT-PCR have played very important role and have been extensively used in infectious diseases surveillance, especially in emergency diseases because of their good specificity, high sensitivity and quick results. However, it is not uncommon to presenting false positive or false negative results if technological operation or lab environment is not qualified [19-23]. In the present study, the developed quality control panel included samples for assessment of sensitivity, specificity and reproducibility. We designed the panel that included a series of diluted H5N1 virus lysates and in vitro transcribed H5 HA RNA for sensitivity assessment, and seasonal influenza (H1N1, H3N2 and FluB) for cross-specific detection. Known negative samples and an artificial gene RNA were also integrated into the panel for assessment of cross contamination. Additionally, the panel included duplicate samples for assessment of self-reproducibility.

The panel is relatively stability with high reproducibility. Individual, full interpreted, concise and informative reports should be the standard practice when QCA is implemented in networks [24,25]. It is common that RNA samples easily degenerate if involving undependable processing. Therefore the stability and reproducibility of the panel is very important in implementation of panel. In present study, we performed 3 different treatments on the panel. Firstly, to simulate keeping condition and the amount of time needed in the transportation via FedEx, the panel was placed in an ice box for four days. The second treatment consisted of keeping the panel in an ice box for four days then storing at -80°C for three days. This condition was designed to evaluate the effects of samples being delivered and stored over the weekend when processing is unavailable. The final treatment was keeping the panel at 4°C for two weeks to assess unpredictable variation when the panel was implemented. In comparison to the normal storage condition of -80°C, the results suggested that no obvious affection happened on the three treatments as shown by very good CCIA. Additionally, the panel presented very high reproducibility as demonstrated by very good CCIA between different technicians, PCR machines and laboratories.

There were a relatively low number of discrepancies for both concise and reproducibility in this QCA exercise in Chinese avian influenza virus networks. In this implement of QCA, 2/29 laboratories presented fair or moderate rRT-PCR identification for the sample in the developed quality control panel. And 5/29 laboratories presented poor, fair or moderate reproducibility in excise of quality control panel. However, quality assessment is an educational exercise, not a punitive action; its aim is to assist laboratories in their continuous effort towards a higher quality services as we communicated with local labs before the implementation of assessment. Therefore, it was rather discouraging that those laboratories with inaccurate sizing results did not participate the following years. To be opposite, in this quality control exercise, these laboratories participated a specific training for the diagnosis of the avian influenza virus H5N1 after the QCA. In addition, to our knowledge, it is very difficult to develop a standard cut-off value on rRT-PCR. Professional experience should play important role on the dispose of results, especially, when the high Ct value was present. Besides, rRT-PCR should be fluctuated easier than RT-PCR as general knowledge mentioned. To get together, it should be used to explain why the RT-PCR has better CCIA in both cross concordance and self-reproducibility than rRT-PCR in the implement of QCA.

We cannot know if the QCA results reflected the true practices in diagnosis of avian influenza A (H5N1). QCA is voluntary and might be biased towards better performing laboratories as strongly recommended. Besides, QCA samples are always treated by the same way as routine referrals. However, many laboratories have never attributed the large samples or enrolled in the daily sample pool. Thus, the error rates found could still be overestimated on true laboratory performance.

## Conclusion

We developed a quality control panel of RT-PCR and rRT-PCR for avian influenza virus H5N1 surveillance. The panel showed relatively good stability and high reproducibility which is possible for the implement of the panel. An availably statistical data, which are used to assess the detection capability of net works on avian influenza virus (H5N1), can be obtained relatively easily through implementation of the panel on networks.

## Competing interests

The authors declare that they have no competing interests.

## Authors' contributions

RG designed the study, participated and organized in the implement of the study, and drafted the manuscript; YG participated in the design of the study and performed the statistical analysis. LW and SZ carried out the preparation of the panel and participated in the evaluation of the panel; CL, LY and WW participated in the evaluation of the panel; YS conceived of the study, and participated in its design and coordination. All authors read and approved the final manuscript.

## Pre-publication history

The pre-publication history for this paper can be accessed here:

http://www.biomedcentral.com/1471-2334/11/67/prepub
